# Feasibility and Preliminary Effectiveness of Behavioral Activation for Patients with Cancer and Depression in Japan

**DOI:** 10.1089/pmr.2023.0020

**Published:** 2023-06-23

**Authors:** Takatoshi Hirayama, Yuko Ogawa, Yuko Yanai, Akie Shindo, Moeko Tanaka, Shin-Ichi Suzuki

**Affiliations:** ^1^ Department of Psycho-Oncology, National Cancer Center Hospital, Tokyo, Japan.; ^2^ Department of Palliative Care, National Center for Global Health and Medicine, Tokyo, Japan.; ^3^ Department of Psychology and Welfare, Tokyo Metropolitan Children's Medical Center, Tokyo, Japan.; ^4^ Faculty of Human Sciences, Waseda University, Saitama, Japan.

**Keywords:** behavioral activation, cancer patients, depression, psychotherapy, Psycho-Oncology

## Abstract

**Background:**

Though the effectiveness of behavioral activation (BA) for patients with cancer and depression were reported, there is no evidence in Japan.

**Objectives:**

This study aimed at examining the feasibility and preliminary effectiveness of BA for patients with cancer and depression in Japan.

**Methods:**

This pre–post study without a control group was conducted in patients with cancer and depression in Japan. The program completion rate was compared with those of previous studies to examine feasibility. To examine the preliminary effectiveness, outcomes were evaluated four times: before and immediately after the program, and two weeks and three months after the program ended. The primary outcome was the remission rate of depression using the 17-item version of the GRID Hamilton Rating Scale for Depression (HAMD_17_). Secondary outcomes were self-reported depression, anxiety, quality of life, changes in behavior, values, and perceived reward of activity and environmental factors. Pre- and post-program data were compared using paired-samples *t*-tests, and data obtained at four time points were analyzed using one-way repeated-measures analysis of variance.

**Results:**

Of the 68 patients recruited from February 2018 to January 2022, 32 were registered. The completion rate was 75% (24/32), which was similar to previous studies. The total HAMD_17_ score significantly improved after the program. The remission rate of depression was 62.5% (20/32), which was above the defined threshold value (30%). All but two secondary outcomes significantly improved after the program (*p* < 0.05).

**Conclusions:**

The feasibility and preliminary effectiveness of BA for patients with cancer and depression in Japan were suggested.

The Clinical Trial Registration number: UMIN 000036104.

## Introduction

Psychiatric disorders, including major depression, anxiety disorder, and adjustment disorders, are reported to occur in 30–40% of patients with cancer^1^ and lead to adverse outcomes such as decreased quality of life (QOL), lower adherence to chemotherapy, longer hospitalization, and increased suicide risk.^2–5^ Appropriate treatment of psychiatric disorders in this population is crucial.

Psychological interventions should be considered as the first-line therapeutic approach for depression in patients with a chronic physical health problem, including cancer.^6^ Psychotherapy is effective for patients with cancer and depression, according to a meta-analysis.^7^ There is evidence that patients with cancer may benefit from psychotherapies such as cognitive restructuring-based cognitive-behavioral therapy (CBT) and problem-solving therapy.^8,9^

However, since it is common for patients to experience unpleasant thoughts and feelings regarding their experience with cancer,^10^ many patients with cancer are reluctant to directly face their cancer-related concerns. Further, one common reaction is to attempt to avoid negative feelings. This causes patients to distance themselves from positive aspects of their lives and to lose contact with the very circumstances where the solutions to their emotional problems can be found.^11^ The emotional struggles of patients with cancer appear to be related to behavioral restriction, which reduces exposure to the rewarding and valuable aspects of life.^12^

**FIG. 1.**
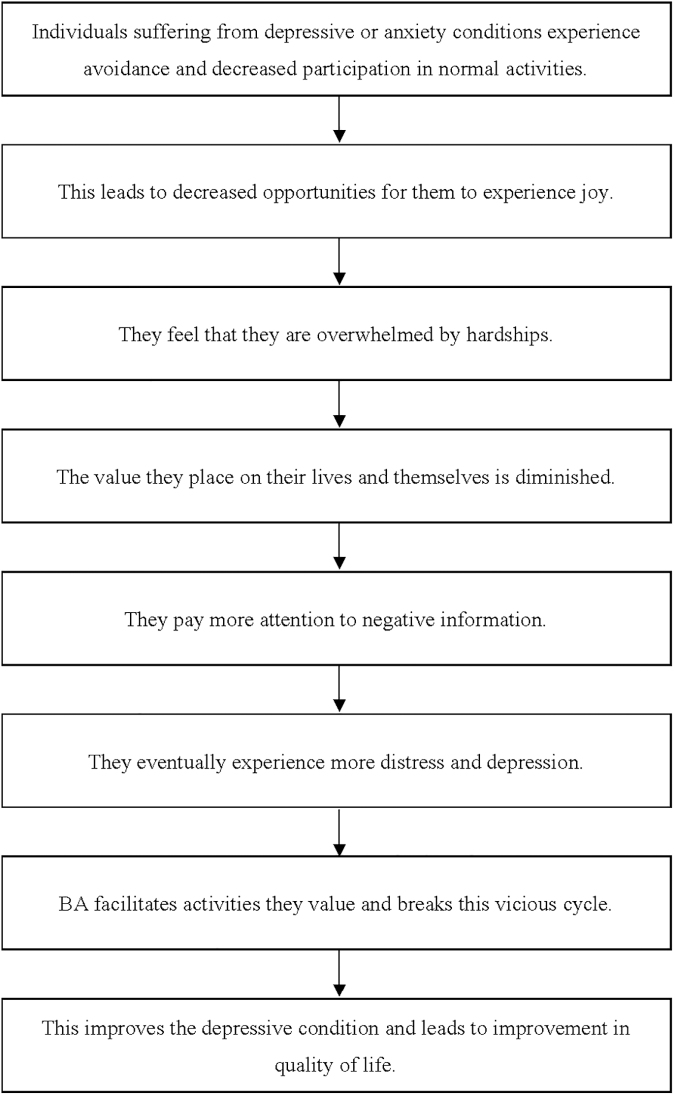
The mechanisms underlying BA. BA, behavioral activation.

Behavioral activation (BA) may be particularly useful for treating the emotional difficulties of patients with cancer, because it emphasizes eliminating avoidance and encouraging involvement in life-giving activities. BA is a psychotherapy that directly approaches patients' most valued daily activities. The mechanisms underlying BA are shown in Figure 1. Notably, BA emphasizes identifying values as a part of behavioral change.^13^ Thus BA, unlike cognitive restructuring-based CBT, is not a cognitive approach and may therefore be useful for cancer patients who are reluctant to talk about their concerns.^14^

The effectiveness of BA for patients with depression is well established.^15^ It is as beneficial as CBT in this population, and is relatively inexpensive, costing 21% less than CBT.^16^ Because the methods of BA are quite simple, the technique can be learned in a short time by medical staff. By standardizing methodology and quality, it may be possible to enhance overall cancer treatment quickly, easily, and at low cost.

Though previous studies have reported the effectiveness of BA for patients with cancer and depression,^17,18^ there is no evidence of BA for them in Japan. Therefore, this study aimed at examining the feasibility and preliminary effectiveness of BA for patients with cancer and depression in Japan.

## Methods

### Study design and procedures

This pre–post study without a control group was conducted to examine the feasibility and preliminary effectiveness of BA for patients with cancer and depression in Japan. The program completion rate was compared with those of previous studies to examine the feasibility. The primary outcome was the remission rate of depression based on the 17-item version of the GRID Hamilton Rating Scale for Depression (HAMD_17_). Secondary outcomes were self-reported depression, anxiety, QOL, changes in behavior, values, and perceived reward of activity and environmental factors. Outcomes were evaluated four times: before and immediately after the program, and at two weeks and three months after the program ended.

The study outline and subject eligibility criteria were announced to each department of the National Cancer Center Hospital in Japan through bulletins, in-hospital emails, and websites. Patients who wished to participate in this study, either on their own initiative or on the recommendation of their attending physician, were evaluated for eligibility in a preliminary interview.

This study was approved by the National Cancer Center Institutional Review Board (approval number, 2017-276) and registered at the UMIN Clinical Trials Registry (UMIN000036104). We obtained written consent from all participants.

### Study participants

The eligibility criteria were as follows: patients with cancer who (1) were undergoing cancer treatment or with a history of cancer; (2) met the diagnostic criteria for major depressive episodes according to the Mini-International Neuropsychiatric Interview^19^; (3) had an HAMD_17_ score of ≥8 (depression of mild severity or worse); (4) were aged 20–64 years or aged ≥65 years with a Mini-Mental State Examination (MMSE)^20^; of ≥24 points; (5) had an Eastern Cooperative Oncology Group performance status score of 0–1, as this facilitates behavior modification; (6) could speak Japanese; and (7) provided written consent to participate in this study.

The exclusion criteria were as follows: (1) serious physical or psychiatric symptoms (cognitive dysfunction, impaired consciousness, severe depression with psychotic symptoms, imminent suicidal ideation, past suicide attempt history) that would prevent program completion; (2) prior intervention by a specialist who conducts BA; and (3) a decision by a researcher or the attending physician of an oncology department that it would be difficult for the individual to participate in the study. For those aged ≥65, or those who did not understand the usual instructions, the MMSE was performed during the preliminary interview, and a score of ≤23 was considered as indicating cognitive dysfunction.^20^

Although this program did not impact patients' regular treatment, special psychotherapies such as BA (conducted elsewhere) and CBT were prohibited. We did not limit or adjust medication use, because some patients required medications in conjunction with psychotherapy to treat depression.

### Sample size

The goal of depression treatment is to achieve remission, which is defined as the near resolution of symptoms.^21^ The remission rate threshold in the current study was 30% and the expected remission rate was 55%, based on the remission rates of 23% (13/56) and 56% (14/25) in the usual treatment group and BA group, respectively, in a study of BA in patients with depression using HAMD_17_ as the endpoint.^22^ In the evaluation of the remission rate based on the HAMD_17_, if the post-program rate was at least 30%, the program was considered useful.

Twenty-five cases were required based on a binomial test that assumed the following: remission rate threshold = 30%, expected remission rate = 0.55, *α* = 0.05 (one side), and 1 − *β* = 0.80. Assuming that 20% of the cases would drop out (i.e., would not complete all seven sessions), we set the sample size at 32 cases.

### BA program

The program consisted of seven 50-minutes sessions that were conducted every 1 to 2 weeks, with an average of 5–10 minutes of homework per day. The themes and contents of the program are shown in Table 1.

**Table 1.** Themes and Contents of the Behavioral Activation Program

Session	Theme	Contents
1	Let's begin!	Understand the relationships between emotions and behaviors Learn tips on how to avoid being preoccupied by cancer to regain pleasure and meaning in life
2	Identify the relationships between emotions and behaviors	Explore the relationships between emotions and daily activities Identify patterns of feeling anxiety and depression and patterns of feeling calm
3	Identify activities that make your life pleasurable	Clarify values in life Identify activities that are achievable in one's current situation
4	Review the results of activities	Evaluate the usefulness and difficulty of each activity Discuss ways to participate in activities that are challenging to engage in
5	Identify difficult situations and understand ways to change one's feelings	Identify situations that are likely to lead to anxiety and depression Identify thoughts that occur in the early stages of anxiety and depression, and any vicious cycle that may be involved Identify pros and cons of negative thinking
6	Learn to live a life of value	Review how the program helped change patterns of daily life Identify future goals and make plans to achieve them
7	Wrap-up and graduation	Review what has been learned through the program Identify future issues and how to address them

The program was verified in a preliminary study that explored the applicability of BA to patients with cancer.^14^ The results showed that the program completion rate and remission rate of depressive symptoms were high regardless of cancer stage.

All therapists were required to be psychotherapists or psychiatrists with clinical experience, including work with patients with cancer, and with sufficient experience in conducting BA, either in the preliminary study^14^ or in BA training. Trainees observed seven BA sessions conducted in a clinical setting by a supervisor with sufficient experience with BA. After observing these sessions, the trainees conducted the BA on their own, although under supervision. Thereafter, the trainees were certified as having sufficient experience to conduct BA.

### Measures

The program completion rate (the number of subjects who completed all seven sessions divided by the number of subjects who participated in the program, multiplied by 100) was calculated to examine feasibility.

The primary outcome was the remission rate among patients with depression, as determined by the HAMD_17_. Secondary outcomes were the Beck Depression Inventory-II (BDI-II), Generalized Anxiety Disorder-7 (GAD-7), Functional Assessment of Cancer Therapy-General (FACT-G), Behavioral Activation for Depression Scale-Short Form (BADS-SF), Valuing Questionnaire (VQ), and Reward Probability Index (RPI). The individuals responsible for measuring outcomes and assessing patients differed from those responsible for conducting the program.

The HAMD_17_ is an amended version of the original Hamilton Depression Rating Scale that provides standardized explicit scoring conventions with a structured interview guide for administration and scoring.^23,24^ The severity of major depressive symptoms was assessed using the HAMD_17_ score, as follows: 0–7, no depression; 8–16, mild depression; 17–23, moderate depression; and ≥24, severe depression.^25^

The BDI-II measures depressive symptoms and consists of 21 self-reported items scored on a 4-point scale.^26^ The psychometric criteria for the BDI-II are generally considered excellent when the instrument is administered to outpatients.^26,27^ Good reliability and validity have been reported for the Japanese version.^28^

The GAD-7 is a seven-item questionnaire developed to identify probable cases of GAD and to measure the severity of GAD symptoms^29^; a Japanese version has been validated.^30^ The total score of the GAD-7 ranges from 0 to 21.

The FACT-G was used to evaluate QOL.^31^ This widely used questionnaire consists of 27 items, with a higher score indicating better QOL. The questionnaire comprises four domains: physical, social, emotional, and functional well-being.

The BADS-SF was developed to assess behavioral changes resulting from BA.^32^ The BADS-SF comprises subscales regarding two traits, Activation and Avoidance. The Japanese version of this scale consists of eight items, one less than in the original version, and the validity and reliability have been confirmed.^33^

The VQ is a 10-item self-reported questionnaire that measures the consistency of one's behaviors with their values.^34^ Scores for each VQ item were calculated for each of the two factors, VQ Progress (VQ-P) and VQ Obstruction (VQ-O). The VQ-P measures the extent to which individuals are aware of what is personally important to them and their perseverance in achieving whatever this is. The VQ-O measures the extent to which living according to one's values is disrupted by avoiding experiences that distract from this goal, either through neglect or by focusing on other psychological experiences. The validity and reliability of the Japanese version have been confirmed.^35^

The RPI was used to assess environmental reward.^36^ The Japanese version of the RPI consists of 19 items (one fewer than the original scale) across three factors: Amount of Reward, Environmental Suppressors, and Reward Skill. The validity and reliability of the Japanese version have been confirmed.^37^

### Data analysis

#### Feasibility

In two previous studies of BA for patients with cancer, the completion rates were 76.2% (32/42)^17^ and 77.3% (17/22),^18^ respectively. In this study, BA was considered feasible if the completion rate was at least 75%. The program completion rate was calculated using the number of program participants as the denominator and the number of people who completed all seven sessions as the numerator.

#### Major statistical analysis

To clarify the preliminary effectiveness of BA for depression in patients with cancer, the HAMD_17_-based remission rate was determined as follows: (the number of patients with an HAMD_17_ score of ≤7 points at the end of the program)/(the number of patients who participated in the program [including dropouts]). The remission rate was evaluated by a binomial test; if the remission rate after program completion was at least 30%, which was the value defined as the threshold, BA was considered useful.

All assessors received extensive HAMD_17_ training. The reliability of the interview ratings was determined by 13 random samples (10.2%), and the interrater agreement (kappa) value for the diagnosis of depression was 1.0, indicating excellent interrater reliability.

#### Secondary statistical analysis

Exploratory secondary statistical analysis was performed to supplement the main statistical analysis results. Quantitative data were analyzed using IBM SPSS Statistics version 28 (IBM Corp, Armonk, NY). The significance level was 5% on both sides. For the BDI-II and GAD-7, the total score of each scale was calculated. For the FACT-G, BADS-SF, VQ, and RPI, each subscale and total score were calculated.

For parametric data, comparisons of data obtained before and immediately after the end of the program (two time points) were analyzed using paired-samples *t*-tests, and data at four time points (before and immediately after the program, and two weeks and three months after the program ended) were analyzed using one-way repeated-measures analysis of variance. For nonparametric data, the Wilcoxon signed-rank test was used to compare pre- and post-program responses.

To ease interpretation, Hedge's *g* was computed, such that values of 0.2, 0.5, and 0.8 denoted small, moderate, and large effect sizes, respectively.^38^

## Results

### Participants

**FIG. 2.**
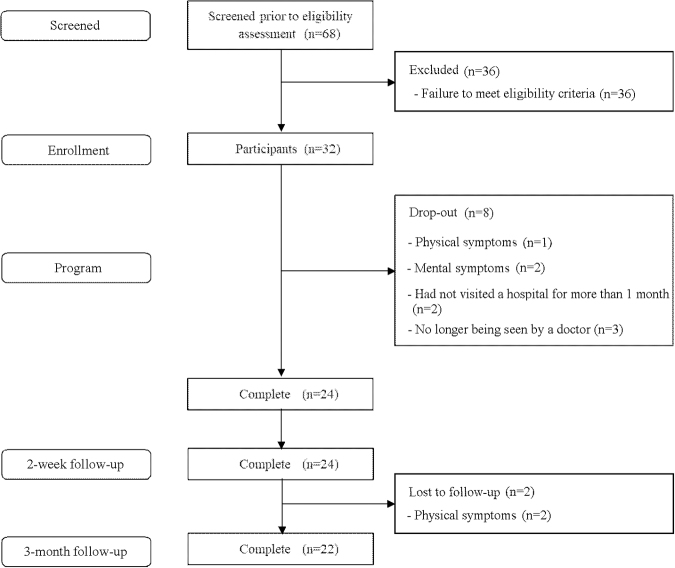
CONSORT Diagram. CONSORT, Consolidated Standards of Reporting Trials.

Participants were recruited from February 2018 to January 2022. Of the 68 patients who were initially recruited, 32 were registered in this study, and 24 completed it (completion rate, 75% [24/32]) (Fig. 2). Thirty-six patients were not registered, because they did not meet the diagnostic criteria for major depressive episodes according to the Mini-International Neuropsychiatric Interview.

The demographic characteristics of patients who completed this program are shown in Table 2. Eighteen females and 6 males with an average age of 55.9 years (SD 10.0) were included. Cancer types included breast cancer (*n* = 10, 41.7%), lung cancer (*n* = 2, 8.3%), colon cancer (*n* = 2, 8.3%), retroperitoneal tumor (*n* = 2, 8.3%), and other (*n* = 8, 33.3%). The most common stage at diagnosis was stage 4 (*n* = 10, 41.7%), followed by stage 1 (*n* = 8, 33.3).

**Table 2.** Demographic Characteristics of Patients Who Completed the Program

	*N*	%
Mean age, years (SD)	55.9	(10.0)
Gender		
Female	18	75.0
Male	6	25.0
Cancer type (primary site)		
Breast	10	41.7
Lung	2	8.3
Colon	2	8.3
Retroperitoneal	2	8.3
Bone and soft tissue	1	4.2
Hematological and lymph	1	4.2
Head and neck	1	4.2
Thymus	1	4.2
Ovary	1	4.2
Renal pelvis	1	4.2
Bladder	1	4.2
Unknown	1	4.2
Disease stage		
1	8	33.3
2	2	8.3
3	2	8.3
4	10	41.7
Recurrence	1	4.2
Unknown	1	4.2
Performance status		
0	23	95.8
1	1	4.2
Past treatment^a^	20	83.3
Surgery	17	70.8
Chemotherapy	14	58.3
Radiation	7	29.2
Current treatment	11	45.8
Chemotherapy	10	41.7
Radiation	1	4.2
Scheduled treatment^a^	15	62.5
Chemotherapy	11	45.8
Radiation	3	12.5
Surgery	2	8.3
Pharmacotherapy^a^	17	70.8
Anxiolytics^a^	10	41.7
Alprazolam	8	
Lorazepam	2	
Etizolam	1	
Antidepressants^a^	9	37.5
Mirtazapine	5	
Trazodone	3	
Sertraline	1	
Escitalopram	1	
Duloxetine	1	
Sleeping pills^a^	8	33.3
Zolpidem	6	
Triazolam	1	
Flunitrazepam	1	
Nitrazepam	1	
Antipsychotics	1	4.2
Aripiprazole	1	
Education status		
College	10	41.7
High school	8	33.3
Junior college	4	16.7
Vocational school	2	8.3
Social status		
Unemployed	15	62.5
Employed	9	37.5
Living		
With someone else	21	87.5
Alone	3	12.5
Marital status		
Married	21	87.5
Unmarried	3	12.5
Parent		
Yes	17	70.8
No	7	29.2
Past psychiatric history		
No	15	62.5
Yes	9	37.5

^a^ Including duplicates.

SD, standard deviation.

### Primary outcome

The total HAMD_17_ score significantly improved after the program with large effect sizes (Hedge's *g* = 1.95) (Tables 3 and 4). The remission rate of depression was 62.5% (20/32). BA was considered useful, because the remission rate after the program exceeded the pre-defined threshold of 30%.

**Table 3.** Score Differences Before and After the Program (Paired *t*-Test, Two-Sided Test)

	Pre		Post		*t*	*P*	Hedge's g
	*M*	SD	*M*	SD			
HAMD_17_	17.9	5.2	4.4	4.9	9.9	<0.001	1.95
BDI-II	29.0	9.0	16.0	10.9	5.4		1.06
GAD-7	11.9	5.3	6.1	4.9	6.2		1.23
FACT-G							
Physical	13.3	6.2	17.1	8.1	−2.9	0.008	−0.58
Social/Family	13.0	6.4	16.0	6.2	−3.2	0.004	−0.63
Emotional	8.5	5.0	13.2	5.5	−5.0	<0.001	−0.98
Functional	8.5	3.6	13.9	5.4	−5.9		−1.17
Total	43.3	12.7	60.3	19.3	−5.4		−1.06
VQ Obstruction	15.1	4.4	14.5	5.2	0.6	0.575	0.11
BADS-SF							
Activation	6.2	3.4	11.9	5.0	−6.1	<0.001	−1.19
Avoidance^a^	12.7	3.4	15.0	3.4	−3.8		−0.75
Total	18.9	4.9	27.0	6.4	−6.2		−1.23
RPI Environmental Suppressors^a^	22.5	3.8	24.8	3.7	−2.8	0.010	−0.55

^a^ Reverse-scaled items.

BADS-SF, Behavioral Activation for Depression Scale-Short Form; BDI-II, Beck Depression Inventory-II; FACT-G, Functional Assessment of Cancer Therapy-General; GAD-7, Generalized Anxiety Disorder-7; HAMD_17_, the 17-item version of the GRID Hamilton Rating Scale for Depression RPI, Reward Probability Index; VQ, Valuing Questionnaire.

**Table 4.** Score Differences Before and After the Program (Wilcoxon Signed-Rank Test, Two-Sided Test)

	Pre		Post		*W*	*p*	*r*
	*M*	IQR	*M*	IQR			
VQ Progress	11.0	6.8	15.0	8.8	249.0	<0.001	0.81
RPI							
Amount of Reward	14.0	4.0	15.5	4.8	187.5	0.002	0.63
Reward Skill	6.0	2.0	6.0	2.8	42.5	0.120	0.31
Total	43.5	10.3	47.0	7.5	218.5	0.003	0.61

### Secondary outcomes

Regarding changes before and after the program, normality was confirmed for all outcomes except VQ-P, RPI Amount of Reward, RPI Reward Skill, and RPI Total.

All secondary outcomes except VQ-O and RPI Reward Skill were significantly improved immediately after the program (*p* < 0.05) (Tables 3 and 4) and three months later (Tables 5 and 6). Two patients dropped out from the study due to mental symptoms. The pre-scores of HAMD_17_ for them were 24 and 31, respectively.

**Table 5.** One-Way Repeated-Measures Analysis of Variance

	Pre		Post		Two-week follow-up		Three-month follow-up		*F*	*p*	Partial η^2^	Multiple comparison
	*M*	SD	*M*	SD	*M*	SD	*M*	SD				
HAMD_17_	17.5	5.2	4.1	4.5	5.0	5.0	5.9	7.3	46.50	<0.001	0.69	Pre > post, two-week, three-month
BDI-II	29.1	9.3	15.9	11.4	15.7	11.4	14.6	12.2	24.02		0.53	
GAD-7	12.2	5.3	6.4	5.0	6.8	5.3	6.5	5.4	22.11		0.51	
FACT-G												
Physical	13.1	6.2	17.4	8.4	18.0	7.8	18.9	7.9	10.91	<0.001	0.34	Pre < post, two-week, three-month
Social/Family	13.2	6.6	16.2	6.5	13.7	7.7	15.9	6.8	3.75	0.015	0.15	Pre < post, three-month
Emotional	8.3	5.1	13.1	5.7	13.8	5.5	13.5	5.3	18.98	<0.001	0.48	Pre < post, two-week, three-month
Functional	8.7	3.6	14.5	5.2	13.8	4.8	15.4	5.9	24.18		0.54	
Total	43.2	13.1	61.1	19.8	59.2	19.8	63.7	20.8	20.31		0.49	
VQ Obstruction	15.3	4.4	15.0	5.1	14.6	4.8	14.3	5.2	0.42	0.738	0.02	—
BADS-SF												
Activation	6.4	3.3	12.3	5.1	12.0	5.7	11.7	6.1	12.13	<0.001	0.37	Pre < post, two-week, three-month
Avoidance^a^	12.7	3.4	14.9	3.5	14.2	3.4	14.6	3.1	6.45		0.24	
Total	19.1	5.1	27.2	6.6	26.2	7.8	26.3	7.7	15.54		0.43	
RPI Environmental Suppressors^a^	22.5	4.0	24.6	3.8	25.1	4.2	25.9	4.0	6.94		0.25	

^a^ Reverse-scaled items.

**Table 6.** Friedman Repeated-Measures Analysis of Variance on Ranks

	Pre		Post		Two-week follow-up		Three-month follow-up		χ^2^	*p*	*r*	Multiple comparison
	*M*	IQR	*M*	IQR	*M*	IQR	*M*	IQR				
VQ Progress	11.0	7.5	15.0	9.0	16.5	7.5	14.0	5.8	31.41	<0.001	0.81	Pre > post
											0.77	Pre >2-week
											0.86	Pre >3-month
RPI												
Amount of Reward	14.0	4.5	16.0	5.5	16.5	5.0	17.0	4.0	14.3	0.002	0.63	Pre >3-month
Reward Skill	6.0	2.0	6.0	3.0	6.0	2.0	6.0	2.5	2.79	0.436	—	—
Total	43.5	11.5	47.0	8.5	49.0	9.3	47.0	9.8	15.64	<0.001	0.65	Pre >2-week
											0.67	Pre >3-month

ANOVA, analysis of variance.

## Discussion

This is the first study to examine the feasibility and preliminary effectiveness of BA for patients with cancer and depression in Japan. The program completion rate was 75% (24/32). This result is consistent with two previous studies in patients with cancer^17,18^ and suggest that BA might be feasible in Japan. Over half of the recruited patients were not registered, because they did not meet the diagnostic criteria for major depressive episodes. Recent surveys in Japan reported that 9.0% of patients with cancer had depressive symptoms.^39^ This low prevalence of depression might have contributed to the low registration rate.

Because the remission rate of depression was 62.5% (≥30%), this program was considered useful. This remission rate is similar to that of 56% achieved by Dimidjian et al. in a study of BA for patients with depression using HAMD_17_ as the endpoint.^22^ On the other hand, Hopko et al. reported that the HAMD_17_-based remission rate in patients with breast cancer was 71%.^17^ Both the current study and that of Hopko et al. had similar HAMD_17_ scores before the program (17.9 [5.2] and 19.2 [7.0], respectively).

The discrepancy in remission rates may reflect the differences between populations regarding cancer stages; for instance, 41.7% of patients in this study had stage 4 disease, compared with 1% of patients in the study by Hopko et al. Many of the subjects in this study were stage 1 and 4. Remission rates for stage 1 and stage 4 patients were similar at 87.5% (7/8) and 80% (8/10), respectively. Our findings suggest that BA might also be effective in patients with advanced cancer.

HAMD_17_ scores remained low two weeks and three months after the program ended. These results suggest that the effectiveness of BA on depression continues for at least three months after the program ended.

All secondary outcomes except VQ-O and RPI Reward Skill were significantly improved after the program (*p* < 0.05). VQ-P measures the extent to which individuals are aware of what is personally important to them and their perseverance in achieving it. BA emphasizes identifying values as a part of behavioral change,^13^ and this may improve VQ-P rather than VQ-O.

Our findings suggested that documenting activities and supporting value-oriented behavior in BA improved RPI Amount of Reward and RPI Environmental Suppressors. Awareness of the vicious cycle of activity-suppressive living might increase activity and improve depression, and expansion of value-oriented behaviors might lead to improved QOL.

In previous randomized studies of BA for patients with cancer, BDI-II and BADS were significantly improved after the intervention.^17,18^ Although the scales used in this study were different, BA improved anxiety, health-related QOL, and positive or pleasurable outcomes or rewards that follow behaviors. Our findings are consistent with these results, and BA may not only improve depression in patients with cancer, but also provide broad support to help patients better deal with the cancer trajectory and lead a more fulfilling life based on their values. This suggests that BA may supplement psychotherapy as a comprehensive support for patients with cancer.

This study has several limitations. First, it used a single-arm pre–post design without a control arm, which may have caused several systematic biases. To examine the effectiveness of BA, a randomized controlled trial is needed as a next step. Second, this study was conducted at a single cancer center, raising the question of institutional bias, and the results may not be applicable to other settings. Third, nearly half of the patients had breast cancer.

Breast cancer patients were reported to have psychological distress, especially fear of cancer recurrence.^40^ Though the needs of breast cancer patients were high, this study suggested the clinical applicability of BA to other cancer patients. Fourth, this study did not reach the target registry due to dropouts, so the results should be interpreted with caution. The introduction of BA for severely depressed patients with cancer should be carefully considered, as high-scoring patients have dropped out due to psychiatric symptoms.

Finally, the preliminary effectiveness of BA should not be exaggerated due to the possibility of bias, such as the effect of pharmacotherapies and other co-interventions (support from medical staff and their family members). In this study, about 70% of the participants had pharmacotherapies, including antidepressants.

The effect of pharmacotherapies should be carefully considered, since a systematic review reported low-certainty evidence for antidepressants compared with placebo in the treatment of depression in patients with cancer.^41^ This study is a pilot study and will be conducted with a more inclusive population as the next step.

## Conclusions

This study suggested the feasibility and preliminary effectiveness of BA for patients with cancer and depression in Japan.

## Data Availability

All data generated or analyzed during this study are included in this published article.

## Abbreviations Used

BA: behavioral activation
BADS-SF: Behavioral Activation for Depression Scale-Short Form
BDI-II: Beck Depression Inventory-II
CBT: cognitive-behavioral therapy
CONSORT: Consolidated Standards of Reporting Trials
FACT-G: Functional Assessment of Cancer Therapy-General
GAD-7: Generalized Anxiety Disorder-7
HAMD_17_: Hamilton Rating Scale for Depression
MMSE: Mini-Mental State Examination
PS: performance status
QOL: quality of life
RPI: Reward Probability Index
VQ: Valuing Questionnaire
VQ-O: VQ Obstruction
VQ-P: VQ Progress
